# A Dynamic Model for Prediction of Psoriasis Management by Blue Light Irradiation

**DOI:** 10.3389/fphys.2017.00028

**Published:** 2017-01-26

**Authors:** Zandra C. Félix Garza, Joerg Liebmann, Matthias Born, Peter A. J. Hilbers, Natal A. W. van Riel

**Affiliations:** ^1^Department of Biomedical Engineering, Eindhoven University of TechnologyEindhoven, Netherlands; ^2^Philips Technologie GmbH, Innovative TechnologiesAachen, Germany

**Keywords:** inflammatory skin conditions, epidermis, keratinocytes, computational model, phototherapy, visible light

## Abstract

Clinical investigations prove that blue light irradiation reduces the severity of psoriasis vulgaris. Nevertheless, the mechanisms involved in the management of this condition remain poorly defined. Despite the encouraging results of the clinical studies, no clear guidelines are specified in the literature for the irradiation scheme regime of blue light-based therapy for psoriasis. We investigated the underlying mechanism of blue light irradiation of psoriatic skin, and tested the hypothesis that regulation of proliferation is a key process. We implemented a mechanistic model of cellular epidermal dynamics to analyze whether a temporary decrease of keratinocytes hyper-proliferation can explain the outcome of phototherapy with blue light. Our results suggest that the main effect of blue light on keratinocytes impacts the proliferative cells. They show that the decrease in the keratinocytes proliferative capacity is sufficient to induce a transient decrease in the severity of psoriasis. To study the impact of the therapeutic regime on the efficacy of psoriasis treatment, we performed simulations for different combinations of the treatment parameters, i.e., length of treatment, fluence (also referred to as dose), and intensity. These simulations indicate that high efficacy is achieved by regimes with long duration and high fluence levels, regardless of the chosen intensity. Our modeling approach constitutes a framework for testing diverse hypotheses on the underlying mechanism of blue light-based phototherapy, and for designing effective strategies for the treatment of psoriasis.

## Introduction

Blue light (BL) at 453 nm is non-toxic (Awakowicz et al., 2009; Kleinpenning et al., 2010) and decreases the symptoms of psoriasis vulgaris (Pv) (Weinstabl et al., 2011; Pfaff et al., 2015), a chronic, inflammatory skin condition that affects 2–3% of the world's population (Parisi et al., 2013). Psoriasis vulgaris is characterized by hyper proliferation and lowered differentiation of skin keratinocytes (Weinstein et al., 1985), evident in lesional areas of thick skin (Perera et al., 2012). These areas also exhibit sustained inflammation caused by immune cells such as T cells and dendritic cells (Perera et al., 2012). Blue light minimizes the proliferation of keratinocytes and induces their differentiation in a wavelength and fluence dependent manner (Liebmann et al., 2010). Additionally, blue light irradiation suppresses dendritic cell activation (Fischer et al., 2013). These effects of blue light on keratinocytes and immune cells may explain the reduced inflammation and diminished epidermal thickness of lesional psoriatic skin after the treatment. Nevertheless, the underlying mechanism of this therapeutic approach is not fully understood. It is not clear how the cellular processes of proliferation and differentiation are modified in the cells after irradiation with blue light. Further, it is uncertain whether blue light affects only proliferative cells or both proliferative and non-proliferative cells. It is hypothesized that BL improves psoriatic skin by decreasing the proliferative capacity of keratinocytes.

Large variations are observed in the results from the available clinical investigations (Maari et al., 2003; Weinstabl et al., 2011; Kleinpenning et al., 2012; Pfaff et al., 2015). These discrepancies in the reported outcome may be due to differences in the main treatment parameters, i.e. length of treatment (days), fluence *F* (Jcm^−2^) also referred to as dose, and power density (mWcm^−2^) also denoted as intensity. Additionally, a BL treatment session may occur one or more times per week for a certain number of weeks (Maari et al., 2003; Weinstabl et al., 2011). Despite all the potential combinations of treatment parameters and their impact on the effectiveness of the treatment, the effect of variations in the irradiation parameters of BL on the dynamics of keratinocytes is unknown. Additionally, no clear guidelines have been defined in the literature for BL treatment of psoriasis. The current treatments for psoriasis include phototherapy with ultraviolet (UV) light (Schneider et al., 2008). UV phototherapy is effective but only indicated for severely affected patients due to the risk of skin cancer (Pathak, 1991) caused by DNA damage. In contrast with UV, blue light is not toxic to skin cells (Awakowicz et al., 2009). The difference in the therapeutic effects of both spectral ranges is the result of the interactions between these spectral ranges and their molecular photoacceptors. UV is absorbed by DNA (Markovitsi, 2016) and yields DNA damage (Lee et al., 2013), cell death, and remission of psoriasis. Blue light is accepted by flavins (Eichler et al., 2005) and porphyrins (Dai et al., 2012), resulting in a decreased proliferative capacity and management of psoriasis. The non-toxicity and beneficial effects of blue light make it an attractive alternative to UV phototherapy. Emphasizing the need for establishing clear treatment recommendations that lead to an effective therapeutic regime using blue light.

Considering that the details of the mechanism of blue light-based therapy for psoriasis are still not fully elucidated, it seems suitable to use computational methods for their analysis. Computational models have previously been used to predict cellular behaviors (Savill, 2003; Gandolfi et al., 2011) and the effects of ultraviolet irradiation on the skin (Weatherhead et al., 2011; Zhang et al., 2015). This approach may contribute to the investigation of processes occurring in the epidermis after blue light irradiation, and extend our knowledge on the principles of regulatory effects induced by this spectral range on the keratinocytes dynamics.

Here, we study the blue light treatment of psoriasis using an *in silico* approach. We first used the model to explore whether a temporary decrease of keratinocytes hyper-proliferation can explain the outcome of phototherapy with blue light. The model accurately described the response to blue light therapy. The simulations suggested that the observed decrease in the keratinocytes proliferation rate is sufficient to reduce the epidermal thickness and severity of psoriasis. However, it was not sufficient to allow psoriatic epidermis to completely remodel back to a healthy phenotype, regardless the treatment scheme. Then, we analyzed the effect of length of treatment, fluence, and intensity on the management of this inflammatory skin condition. The model predicted that high efficacy is achieved by treatment schemes with long duration and high fluence levels.

## Materials and methods

**Figure 1:**
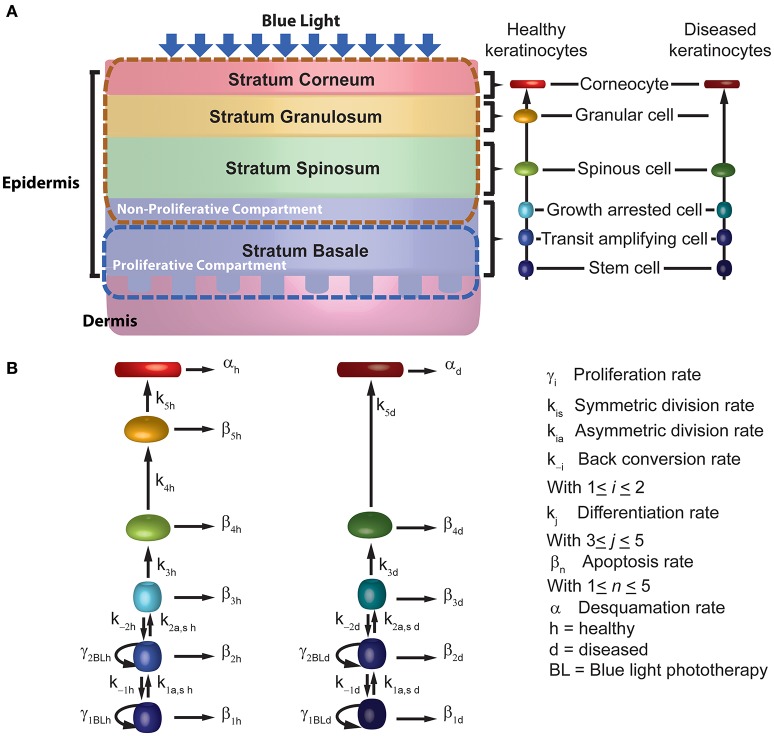
**Schematic description of the mechanistic model for blue light treatment of psoriasis. (A)** The model considers the 4 sub-layers of the epidermis, the 6 stages of differentiation for keratinocytes across the sub-layers. **(B)** BLISS accounts for the cellular processes of proliferation (γ), differentiation (*k*), apoptosis (β), and desquamation (α).

To describe the management of psoriasis by blue light, we implemented a computational model for BL irradiation of psoriatic skin (BLISS; Figure 1). This model is defined by a set of 12 ordinary differential equations (ODEs) describing the time evolution of keratinocytes as they move vertically through the layers of the epidermis while blue light is shined upon them. BLISS was developed based on the phenomenological observations of decreased proliferation and increased differentiation of keratinocytes due to blue light, particularly at a wavelength of 453 nm. In this section we describe the general structure of the model, its implementation, and analysis.

### Computational model

In contrast with UV, BL does not lead to cell death below fluences of 500 Jcm^−2^ (Awakowicz et al., 2009). Instead, it affects the proliferation and differentiation of the keratinocytes, which are key processes in the model we propose. The general structure and assumptions considered in the model regarding the kinetics of keratinocytes in psoriasis are based on the work of Zhang et al. (2015) for UV phototherapy of psoriasis. However, the main difference with their model is the implementation of the underlying mechanism by which the kinetics of the keratinocytes are affected upon BL irradiation.

In the model (Figure 1), we represent psoriasis as a bi-stable system and assume that keratinocytes show either a healthy or diseased phenotype. We consider that both populations coexist and interact within the epidermis. This assumption allows the description of the effects induced by blue light therapy. The set of ODEs describing the interactions between healthy and diseased keratinocytes under the influence of blue light are presented by Equations (1–12). This set of equations describes the kinetics of keratinocytes at six stages of differentiation in both their healthy (Equations 1–6) and diseased (Equations 7–12) state, i.e., stem cells (*P*_sch, d_), transit amplifying cells (P_ah, d_), growth arrested cells (P_gah, d_), spinous cells (P_sph, d_), granular cells (P_gch, d_), corneocytes (P_cch, d_). Stem cells and transit amplifying cells form the proliferative compartment of the epidermis, and corneocytes are the end point of the differentiation process. The ODEs account for the cellular processes of proliferation (γ_h, d_), differentiation (k_h, d_), apoptosis (β_h, d_), and desquamation (α_h, d_). The cells in the proliferative compartment may divide in one of three modes, i.e., produce to daughter cells equal to the progenitor [self-proliferation (γ_*h, d*_)], generate two daughter cells where one corresponds to the next stage of differentiation [asymmetric division (*k*_*ah, d*_)], or induce two daughter cells where both correspond to the next stage of differentiation [symmetric division (*k*_*sh, d*_)]. The description of each parameter considered in these equations is presented in Table 1. These parameters were derived from one of three sources, i.e., the literature, calculated from other model parameters as specified in Table 1, or estimated by fitting the model to experimental data of Liebmann et al. (2010).

(1) $$\begin{array}{l} \frac{dP_{sch}}{dt}=\left[\gamma_{1h}\theta_{BL\gamma }\left(1-\frac{P_{sch}+\frac{P_{scd}}{\lambda }}{P_{sc}^{max}}\right)-k_{1sh}\theta_{BLk}-\beta_{1h}\right]\\ P_{sch}+k_{-1h}\;P_{tah} \end{array}$$

(2) $$\begin{array}{l} \frac{dP_{tah}}{dt}=\left(\gamma_{2h}\theta_{BL\gamma }-k_{2sh}\theta_{BLk}-\beta_{2h}-k_{-1h}\right)\\ P_{tah}-\left(k_{1ah}+2k_{1sh}\right)\theta_{BLk}P_{sch}+k_{-2h}P_{gah} \end{array}$$

(3) $$\begin{array}{l} \frac{dP_{gah}}{dt}=\left(k_{2ah}+k_{2sh}\right)\theta_{BLk}\\ P_{tah}-\left(k_{-2h}+k_{3h}\theta_{BLk}+\beta_{3h}\right)P_{gah} \end{array}$$

(4) $$\begin{array}{l} \frac{dP_{sph}}{dt}=k_{3h}\theta_{BLk}P_{gah}-\left(k_{4h}\theta_{BLk}+\beta_{4h}\right)P_{sph} \end{array}$$

(5) $$\begin{array}{l} \frac{dP_{gch}}{dt}=k_{4h}\theta_{BLk}P_{sph}-\left(k_{5h}\theta_{BLk}+\beta_{5h}\right)P_{gch} \end{array}$$

(6) $$\begin{array}{l} \frac{dP_{cch}}{dt}=k_{5h}\theta_{BLk}P_{gch}-a_{h}P_{cch} \end{array}$$

(7) $$\begin{array}{l} \frac{dP_{scd}}{dt}=\left[\gamma_{1d}\theta_{BL\gamma }\left(1-\frac{P_{sch}+P_{scd}}{\lambda P_{sc}^{max}}\right)-k_{1sd}\theta_{BLk}-\beta_{1d}\right]\\ P_{scd}-\frac{K_{p}P_{scd}^{2}}{K_{a}^{2}+P_{scd}^{2}}+k_{-1d}P_{tad} \end{array}$$

(8) $$\begin{array}{l} \frac{dP_{tad}}{dt}=\left(\gamma_{2d}\theta_{BL\gamma }-k_{2sd}\theta_{BLk}-\beta_{2d}-k_{-1d}\right)P_{tad}\\ -\left(k_{1ad}+2k_{1sd}\right)\theta_{BLk}P_{scd}+k_{-2d}P_{gad} \end{array}$$

(9) $$\begin{array}{l} \frac{dP_{gad}}{dt}=\left(k_{2ad}+k_{2sd}\right)\theta_{BLk}P_{tad}\\ -\left(k_{-2d}+k_{3d}\theta_{BLk}+\beta_{3d}\right)P_{gad} \end{array}$$

(10) $$\begin{array}{l} \frac{dP_{spd}}{dt}=k_{3d}\theta_{BLk}P_{gad}-\left(k_{4h}\theta_{BLk}+\beta_{4d}\right)P_{spd} \end{array}$$

(11) $$\begin{array}{l} \frac{dP_{gcd}}{dt}=0 \end{array}$$

(12) $$\begin{array}{l} \frac{dP_{ccd}}{dt}=k_{5d}\theta_{BLk}P_{gcd}-a_{d}P_{ccd} \end{array}$$

**Table 1:** **BLISS model parameterization**.

**Parameter**	**Description**	**Value**	**Source**
$P_{sc}^{max}$	Growth capacity of stem cells	4.5 × 10^3^ mm^−2^	Zhang et al., 2015
γ_1hom_	Minimal stem cell self-proliferation rate constant	3.30 × 10^−3^ d^−1^	Zhang et al., 2015
k_1shom_	Minimal symmetric healthy stem cell division rate constant	1.64 × 10^−3^ d^−1^	Clayton et al., 2007; Zhang et al., 2015
k_1ahom_	Minimal asymmetric healthy stem cell division rate constant	1.31 × 10^−2^ d^−1^	Clayton et al., 2007; Zhang et al., 2015
γ_2h_	Healthy transit amplifying cells self-proliferation rate constant	1.40 × 10^−2^ d^−1^	Bauer et al., 2001; Hoath and Leahy, 2003; Zhang et al., 2015
k_2sh_	Healthy transit amplifying cells symmetric division rate constant	1.73 × 10^−2^ d^−1^	Bauer et al., 2001; Hoath and Leahy, 2003; Zhang et al., 2015
k_2ah_	Healthy transit amplifying cells asymmetric division rate constant	1.38 × 10^−1^ d^−1^	Bauer et al., 2001; Hoath and Leahy, 2003; Zhang et al., 2015
k_3h_	Healthy growth arrested to spinous cells differentiation rate constant	2.16 × 10^−1^ d^−1^	Bauer et al., 2001; Hoath and Leahy, 2003; Zhang et al., 2015
k_4h_	Healthy spinous to granular cells differentiation rate constant	5.56 × 10^−2^ d^−1^	Bauer et al., 2001; Hoath and Leahy, 2003; Zhang et al., 2015
k_5h_	Healthy granular cells to corneocytes differentiation rate constant	1.11 × 10^−1^ d^−1^	Bauer et al., 2001; Hoath and Leahy, 2003; Zhang et al., 2015
k_−1h_	Back conversion rate constant of healthy cells (transit amplifying to stem cells)	1.00 × 10^−6^ d^−1^	Zhang et al., 2015
k_−2h_	Back conversion rate constant of healthy cells (growth arrested to transit amplifying cells)	1.00 × 10^−6^ d^−1^	Zhang et al., 2015
ω	Maximum fold increase of stem cells proliferation rate	100	Heenen et al., 1998; Zhang et al., 2015
*n*	Stem cells proliferation rate regulation by transit amplifying cells	3	Zhang et al., 2015
AI*_*h*_*	Epidermal apoptosis index for healthy skin	0.12%	Bauer et al., 2001; Hoath and Leahy, 2003; Zhang et al., 2015
β_1h_	Apoptosis rate of healthy epidermal stem cells	1.97 × 10^−6^ d^−1^	Calculated as described by Equation 16
β_2h_	Apoptosis rate of healthy transit amplifying cells	2.08 × 10^−5^ d^−1^	Calculated as described by Equation 16
β_3h_	Apoptosis rate of healthy growth arrested cells	2.60 × 10^−4^ d^−1^	Calculated as described by Equation 16
β_4h_	Apoptosis rate of healthy spinous cells	6.68 × 10^−5^ d^−1^	Calculated as described by equation 16
β_5h_	Apoptosis rate of healthy granular cells	1.33 × 10^−4^ d^−1^	Calculated as described by Equation 16
α_h_	Healthy corneocytes desquamation rate constant	7.14 × 10^−2^ d^−1^	Weinstein et al., 1985; Bauer et al., 2001; Hoath and Leahy, 2003; Zhang et al., 2015
ρ_sc_	Fold change of stem cells proliferation in psoriasis	4	Weinstein et al., 1985; Zhang et al., 2015
ρ_ta_	Fold change of transit amplifying cells proliferation in psoriasis	4	Weinstein et al., 1985; Zhang et al., 2015
ρ_tr_	Fold change of psoriatic cells transit rate	5	Weatherhead et al., 2011; Zhang et al., 2015
ρ_de_	Fold change of psoriatic corneocytes desquamation	4	Weinstein and Van Scott, 1965; Zhang et al., 2015
λ	Fold change of stem cells growth capacity in psoriasis	3.5	Heenen et al., 1987; Simonart et al., 2010; Zhang et al., 2015
K_p_	Maximum immune response rate	6	Zhang et al., 2015
K_a_	Half-activation of immune system by psoriatic stem cells density	380	Zhang et al., 2015
γ_1d_	Diseased stem cell self-proliferation rate constant	1.16 × 10^−2^ d^−1^	Calculated as the product of γ_1hom_ and ρ_sc_
k_1sd_	Symmetric diseased stem cell division rate constant	5.70 × 10^−3^ d^−1^	Calculated as the product of k_1shom_ and ρ_sc_
k_1ad_	Asymmetric diseased stem cell division rate constant	4.59 × 10^−2^ d^−1^	Calculated as the product of k_1shom_ and ρ_sc_
γ_2d_	Diseased transit amplifying cells self-proliferation rate constant	4.90 × 10^−2^	Calculated as the product of γ_2h_ and ρ_ta_
k_2sd_	Diseased transit amplifying cells symmetric division rate constant	6.06 × 10^−2^ d^−1^	Calculated as the product of k_2sh_ and ρ_ta_
k_2ad_	Diseased transit amplifying cells asymmetric division rate constant	4.83 × 10^−1^ d^−1^	Calculated as the product of k_2ah_ and ρ_ta_
k_3d_	Diseased growth arrested to spinous cells differentiation rate constant	9.72 × 10^−1^ d^−1^	Calculated as the product of k_3h_ and ρ_tr_
k_4d_	Diseased spinous to granular cells differentiation rate constant	2.50 × 10^−1^ d^−1^	Calculated as the product of k_4h_ and ρ_tr_
k_5d_	Diseased granular cells to corneocytes differentiation rate constant	3.89 × 10^−1^ d^−1^	Calculated as the product of k_5h_ and ρ_tr_
AI*_*d*_*	Epidermal apoptosis index for psoriatic skin	0.035%	Bauer et al., 2001; Hoath and Leahy, 2003; Zhang et al., 2015
β_1d_	Apoptosis rate of diseased epidermal stem cells	2.01 × 10^−6^ d^−1^	Calculated as described by Equation 17
β_2d_	Apoptosis rate of diseased transit amplifying cells	2.12 × 10^−5^ d^−1^	Calculated as described by Equation 17
β_3d_	Apoptosis rate of diseased growth arrested cells	3.40 × 10^−4^ d^−1^	Calculated as described by Equation 17
β_4d_	Apoptosis rate of diseased spinous cells	8.76 × 10^−5^ d^−1^	Calculated as described by Equation 17
β_5d_	Apoptosis rate of diseased granular cells	1.36 × 10^−4^ d^−1^	Calculated as described by Equation 17
α_d_	Diseased corneocytes desquamation rate constant	2.50 × 10^−1^ d^−1^	Calculated as the product of α_h_ and ρ_de_
a_γ_	Blue light coefficient for proliferation factor	1	Estimated from Liebmann et al., 2010
b_γ_	Blue light coefficient for proliferation factor	−3.40 × 10^−3^	Estimated from Liebmann et al., 2010
a_k_	Blue light coefficient for differentiation factor	2.46	Estimated from Liebmann et al., 2010
b_k_	Blue light coefficient for differentiation factor	1.94 × 10^−2^	Estimated from Liebmann et al., 2010
c_k_	Blue light coefficient for differentiation factor	3.46	Estimated from Liebmann et al., 2010
θ_*BL β*500−750_	Blue light factor increasing the apoptosis rate at fluences higher than 500 Jcm^−2^ and lower than 750 Jcm^−2^	3.9 × 10^−2^	Awakowicz et al., 2009
θ_*BL β*>750_	Blue light factor increasing the apoptosis rate at fluences higher than 750 Jcm^−2^	5 × 10^−2^	Awakowicz et al., 2009
ξ_abs_	Energy absorbance of the epidermis for a low perfused Caucasian skin	57.9%	Calculated from optical model

Where

(13) $$\begin{array}{l} \frac{\gamma_{1h}}{\gamma_{\overset{`}{\;}1,hom}}=\frac{k_{1ah}}{k_{1a,hom}}=\frac{k_{1sh}}{k_{1s,hom}}=\frac{\omega }{1+\left(\omega -1\right){\left(\frac{P_{tah}\;+\;P_{tad}}{P_{ta,hom}}\right)}^{n}} \end{array}$$

In psoriasis, the increased number of keratinocytes is the result of a hyper-proliferative population of basal cells and a sustained activation of the immune system. In the model, the hyper-proliferative population is represented by the diseased stem cells and transit amplifying cells proliferating at a rate γ_*d*_, which is faster than the proliferative rate of their healthy counterparts. It is assumed that there are a maximum number of stem cells $P_{sc}^{max}$ available in the epidermis, which limits the numbers of healthy and diseased stem cells *P*_*sch,d*._ The diseased stem cells have a larger growth capacity defined by fold increase λ. The sustained activation of the immune system is considered through the removal of diseased stem cells in Equation (7). The sustained immune system response is regulated by the density of diseased stem cells and defined by the maximum killing rate *K*_*p*_ and the half activation of the immune system *K*_*a*_ due to psoriatic stem cells. The immune response is ample when the cell density of diseased cells exceeds the threshold defined by *K*_*a*_.

In Equation (13), γ_1,*hom*_, *k*_1*a,hom*_, *k*_1*s,hom*_ are the homeostatic rate constants for each division process, and *P*_*ta,hom*_ is the homeostatic density of transit amplifying cells (Zhang et al., 2015). The density of the total transit amplifying cells population is the sum of the healthy and diseased groups of transit amplifying cells. The maximum increase in the growth fraction and decrease in the cell cycle time of fast proliferating stem cells is indicated by ω. *n* represents the steepness of the stem cells proliferation rate regulated by the transit amplifying cells population. When the number of healthy transit amplifying cells is low, the proliferation and division rates of the healthy stem cells increase. Alternatively, the healthy stem cell parameter rates are at their minimum values when the population of healthy transit amplifying cells equals its homeostatic population. It is assumed that psoriatic stem cells are not regulated by the population of transit amplifying cells. Thus, they proliferate with rates ρ_*sc*_ fold higher than the healthy stem cells rate constants in homeostasis. The proliferation and differentiation rates of diseased transit amplifying cells (γ_2*d*_, *k*_2*sd*_, and *k*_2*ad*_) are ρ_*ta*_ fold changes higher than the rates of healthy transit amplifying cells. Similarly the diseased cells in the non-proliferative compartment, i.e., *P*_*gad*_ and *P*_*spd*_, differentiate ρ_*tr*_ times faster than their healthy counterparts. The desquamation (α_*d*_) of psoriatic corneocytes is affected by ρ_*de*_. Note that Equation (11) equals to zero, given that granular cells are lost in psoriasis due to abnormal differentiation.

Irradiation with blue light exponentially decreases the proliferation rate of all healthy and diseased proliferative keratinocytes by a fluence dependent factor θ_*BLγ*_ with a value between 0 and 1 (Equation 14).

(14) $$\begin{array}{l} \theta_{BL\gamma }\;=\;a_{\gamma }e^{\left(b_{\gamma }F\right)}\text{ with }a_{\gamma }>0\text{ and }b_{\gamma }<0 \end{array}$$

Based on the keratinocytes behavior experimentally observed (Liebmann et al., 2010), blue light might also directly affect the differentiation rate of the keratinocytes in an exponential manner by a fluence dependent factor θ_*BLk*_ with a value between 0 and 1 (Equation 15). Parameters related to the decrease of the proliferation and increase of differentiation rates were initially estimated by fitting *in vitro* data from Liebmann et al. (2010).

(15) $$\begin{array}{l} \theta_{BLk}\;=\;c_{k}-a_{k}e^{\left(b_{k}F\right)}\text{ with }a_{k},b_{k}\text{ and }c_{k}\;>0 \end{array}$$

Blue light phototherapy is divided into sessions with a certain fluence *F*, defined by the product of the irradiation time and the average intensity *I*_*av*_ at which light is shined on the skin. The treatment sessions can occur one time per day in a daily or spread out basis. In the model we consider that each day of treatment affects the proliferation of the keratinocytes, based on *F* >0. In days where no treatment is provided *F* = 0 and θ_*BLγ*_ = θ_*BLk*_ = 1.

The apoptosis rates of the healthy keratinocytes β_*h*_ (Equation 16) are determined by the differentiation rate of each population (*k*_1*sh*_*, k*_2*sh*_*, k*_3*h*_*, k*_4*h*_*, k*_5*h*_) and the probability of a cell undergoing apoptosis (apoptotic index *AI*_*h*_). The apoptotic index is a common measurement for quantifying the extent of apoptosis for a given cell population. It is defined as the ratio of apoptosis rate to the total out flux. At fluences higher than 500 Jcm^−2^ BL increases the apoptosis of keratinocytes by factor θ_*BLβ*_. Thus, accounting for the cytotoxicity observed only at high fluences of blue light (Awakowicz et al., 2009). Below 500 Jcm^−2^, θ_*BLβ*_ has a value of 0, for *F* between 500 and 750 Jcm^−2^ its value is 0.039, and 0.05 above 750 Jcm^−2^.

(16) $$\begin{array}{l} \beta_{h}=\;\frac{AI_{h}k_{j}}{1-AI_{h}}+\theta_{BL\beta \;}\;j\in \left\{1_{sh},2_{sh},3_{h},4_{h},5_{h}\right\} \end{array}$$

The diseased keratinocytes have an apoptosis rate defined by the apoptotic index of psoriatic cells *AI*_*d*_, the differentiation rate of each population (*k*_1*sd*_*, k*_2*sd*_*, k*_3*d*_*, k*_4*d*_*, k*_5*d*_) and *q*_*BLβ*_. (Equation 17).

(17) $$\begin{array}{l} \beta_{d}=\;\frac{AI_{d}k_{j}}{1-AI_{d}}+\theta_{BL\beta \;}\;j\in \left\{1_{sd},2_{sd},3_{d},4_{d},5_{d}\right\} \end{array}$$

The skin has specific wavelength dependent optical properties that must be considered in the model, i.e., refractive index, absorption coefficient, scattering coefficient, and anisotropy factor (van Gemert et al., 1989). These properties vary per skin layer and skin type (Zonios et al., 2001). BLISS accounts for a low perfused type 1–3 skin with an epidermal energy absorbance level of 57.9%. This value was computed using an unpublished five-layered optical model developed in LightTools (Synopsis). The energy absorbance value defines the amount of BL energy that is absorbed by the epidermis. Skin types 4–6 have a higher energy absorbance, however are not considered in the model.

### Model implementation and analysis

The model was implemented in Matlab (The Mathworks Inc.). The ODE system was solved with ode-solver *ode15s*. The inputs of the model were the BL irradiation parameters (Table 2) and the initial cell density of the 12 keratinocyte populations (Table 3) representing an psoriatic epidermis (Simonart et al., 2010; Zhang et al., 2015). The total keratinocytes cell density in the simulated epidermis at *t* = 0 was of 217,556 cells per mm^2^, which is 2 times higher than that of healthy skin (~100,000 cells per mm^2^; Hoath and Leahy, 2003). BLISS is available at GitHub^1^ and the BioModels Database^2^ (Chelliah et al., 2015) with identifier MODEL1701090001.

**Table 2:** **Blue light irradiation parameters**.

**Parameter**	**Value**
Fluence	90 Jcm^−2^
Irradiation mode	Continuous or pulsed
Irradiation time	1800 s (30 min)
Duration of treatment	84 days

**Table 3:** **Initial cell density distribution (cells per mm^**2**^)**.

**Keratinocytes population**	**Healthy**	**Psoriatic**
Corneocytes	185	77,633
Granular cells	119	0
Spinous cells	238	79,788
Growth arrested cells	61	20,536
Transit amplifying cells	77	32,098
Stem cells	362	6459
Total	1042 + 216514 = 217,556	

A full treatment of blue light was simulated by modifying the proliferation, differentiation, and apoptotic rates on the day of a BL session during the whole treatment. For example, if the treatment sessions occurred three times per week for 8 weeks, the rates were only varying on those specific days during the 8 weeks of treatment. During a treatment session, light can be shined in a continuous (CW) or pulsed (PW) mode. In CW the fluence of blue light, defined as power density *I* multiplied by time *t*, is provided at a constant peak power density *I*_*p*_, which is equal to the average power density *I*_*av*_. In PW, the epidermis is irradiated in short pulses at an *I*_*p*_ higher than *I*_*av*_. The model allows for the simulation of both modes based on a given duty cycle, fluence, and treatment time. These inputs are used to compute the *I*_*p*_ and duration of each peak and define the instants in which the proliferation rates are decreased during the treatment time.

BLISS calculates the cell densities over time, during and after blue light phototherapy for each healthy and diseased keratinocyte population. The model's results can be directly compared to *in vitro* data. However, in psoriasis most clinical data is given in terms of the psoriasis area and severity index (PASI), or its local form, i.e., the local psoriasis severity index (LPSI; Maari et al., 2003; Weinstabl et al., 2011; Pfaff et al., 2015). PASI is a quantitative rating score for measuring the severity of a psoriatic lesion based on the degree of erythema, scaling and induration per anatomic area, i.e., head, trunk, upper, and lower limbs. Each of the three characteristics is graded on a scale from 0 to 4, giving a maximum score of 12 per area based on the percentage of coverage. The maximum PASI is 72, mild psoriasis which is the target of blue light treatment corresponds to a PASI of 0–10. Induration is related to the thickness of the epidermis, and scaliness is related to the status of the Stratum Corneum. Both features are comprised within the model. Thus, assuming an initial LPSI value and a positive correlation between thickness and scaliness with the cell density of keratinocytes, the LPSI is derived from the relative change in the total cell density of keratinocytes as indicated by Equation (18). A similar approach has been previously described in the literature (Ng et al., 2005; Zhou et al., 2010).

(18) $$\begin{array}{l} LPSI_{i}=LPSI_{0}-LPSI_{0}\left(\frac{P_{Tot_{i}\;-\;}P_{Tot_{0}}}{P_{Tot_{0}}}\right)\;where\;i\geq 1 \end{array}$$

Data from the clinical investigation of Pfaff et al. (2015) was used to verify the adequate description of BL treatment of psoriasis by the model.

#### Multiple parameter sensitivity analysis

A multiple parameter sensitivity analysis (MPSA) was performed to understand how the system may be affected by uncertainty in the model's parameters. An initial simulation for blue light treatment was executed with the nominal model parameters θ_*ref*_ presented in Table 1. From this first simulation the reference output response *y*_*ref*_(*k*) was computed, where *k* represents the cell density of each keratinocyte cell densities derived by the model. 500,000 random parameter value sets were generated with the *lhsdesign* MATLAB function. The parameter sets θ*(i)* were used as input to the model and simulations were implemented. The cost function *v*(*i*) for the simulation results obtained with each parameter set was calculated as the sum of squared errors between the perturbed *y*_*sim*_ and reference output *y*_*ref*_ (Equation 19).

(19) $$v(i)\;=\;\sum_{k\;=\;1}^{N}{\left(y_{ref}\left(k\right)-y_{sim}\left(k,\theta (i)\right)\text{​}\right)}^{2}$$

The parameter sets were then classified as acceptable or unacceptable and the cumulative frequency functions were computed for the acceptable and unacceptable values of each parameter. Then, the Kolmogorov–Smirnov statistic was calculated as the separation between *S*_*a*_*(*θ*)* and *S*_*u*_*(*θ*)* (Equation 20).

(20) $$D=max\left|S_{a}(\theta )-S_{q}(\theta )\right|$$

Finally, this value was used to derive the sensitivity of each parameter on the cell density of all keratinocyte populations at each of the six differentiation states and the local psoriasis severity index. Additionally, a local sensitivity analysis (LPSA; Marino et al., 2008) was performed for the parameters identified by the MPSA for further understanding of how small perturbations in these parameters reflect on the output of the model.

## Results

### The regulation of the keratinocytes proliferation is key in the mechanism of blue light irradiation

Given the phenomenological observations reported in the literature and the flexibility offered by the model to describe them, we first analyzed all the possible *in silico* representations of the changes induced on the proliferation and differentiation of the keratinocytes. Table 4 presents a summary of all potential cases.

**Table 4:** **Possible cases describing the blue light effects on the cellular processes of keratinocytes**.

**Case**	**Rate modified**	**Value of blue light factors *θ_*BLγ*_* and *θ_*BLk*_***	**Population affected**
1	Self-proliferation rates *γ_1_* and *γ_2_* are decreased.	θ_*BLγ*_ < 1 and θ_*BLk*_ = 1 for proliferative cells	Proliferative cells, i.e. stem cells and
2	Asymmetric (*k_1*a*_ and k_2*a*_*) and symmetric (*k_1*s*_ and k_2*s*_*) division rates of proliferative cells are increased.	θ_*BLγ*_ = 1 and θ_*BLk*_ > 1 for proliferative cells	transit amplifying cells.
3	The self-proliferation rates (*γ_1_* and *γ_2_*) are decreased and the asymmetric and symmetric division rates (*k_1*a*_, k_2*a*_, k_1*s*_, and k_2*s*_*) are increased.	θ_*BLγ*_ < 1 and θ_*BLk*_ > 1 for proliferative cells	
4	Asymmetric (*k_1*a*_ and k_2*a*_*) division rates are increased.	θ_*BLγ*_ = 1 and θ_*BLk*_ > 1 (for the asymmetric division rates of proliferative cells)	
5	Symmetric (*k_1*s*_ and k_2*s*_*) division rates are increased.	θ_*BLγ*_ = 1 and θ_*BLk*_ > 1 (for the symmetric division rates of proliferative cells)	
6	The self-proliferation rates (*γ_1_* and *γ_2_*) are decreased and the asymmetric division rates (*k_1*a*_* and *k_2*a*_*) are increased.	θ_*BLγ*_ < 1 and θ_*BLk*_ > 1 (for the asymmetric division rates of proliferative cells)	
7	The self-proliferation rates (*γ_1_* and *γ_2_*) are decreased and the symmetric division rates (*k_1*s*_ and k_2*s*_*) are increased.	θ_*BLγ*_ < 1 and θ_*BLk*_ > 1 (for the symmetric division rates of proliferative cells)	
8	Differentiation rates (*k_3_, k_4_*, and *k_5_*) of non-proliferative cells are increased.	θ_*BLγ*_ = 1 and θ_*BLk*_ > 1 (for division rates of non-proliferative cells)	Non-proliferative cells, i.e. growth arrested cells, spinous cells, and granular cells.
9	Differentiation rates (*k_1*a*_, k_1*s*_, k_2*a*_, k_2*s*_, k_3_, k_4_*, and *k_5_*) of all cells are increased.	θ_*BLγ*_ = 1 and θ_*BLk*_ > 1 (for division rates of all cells)	Proliferative cells, i.e. stem cells and transit amplifying cells. Non-proliferative
10	The self-proliferation rates (*γ_1_* and *γ_2_*) are decreased and the differentiation rates (*k_3_, k_4_*, and *k_5_*) of non-proliferative cells are increased.	θ_*BLγ*_ < 1 and θ_*BLk*_ > 1 (for division rates of non-proliferative cells)	cells, i.e. growth arrested cells, spinous cells, and granular cells.
11	The asymmetric division and differentiation rates (*k_1*a*_, k_2*a*_, k_3_, k_4_*, and *k_5_*) are increased.	θ_*BLγ*_ = 1 and θ_*BLk*_ > 1 (for the asymmetric division rates of proliferative cells and the division rates of non-proliferative cells)	
12	The symmetric division and differentiation rates (*k_1*s*_, k_2*s*_, k_3_, k_4_*, and *k_5_*) are increased.	θ_*BLγ*_ = 1 and θ_*BLk*_ > 1 (for the symmetric division rates of proliferative cells and the division rates of non-proliferative cells)	
13	The self-proliferation rates (*γ_1_* and *γ_2_*) are decreased and the division and differentiation rates (*k_1*a*_, k_1*s*_, k_2*a*_, k_2*s*_, k_3_, k_4_*, and *k_5_*) are increased.	θ_*BLγ*_ > 1 and θ_*BLk*_ > 1 (for division rates of all cells)	

**Figure 2:**
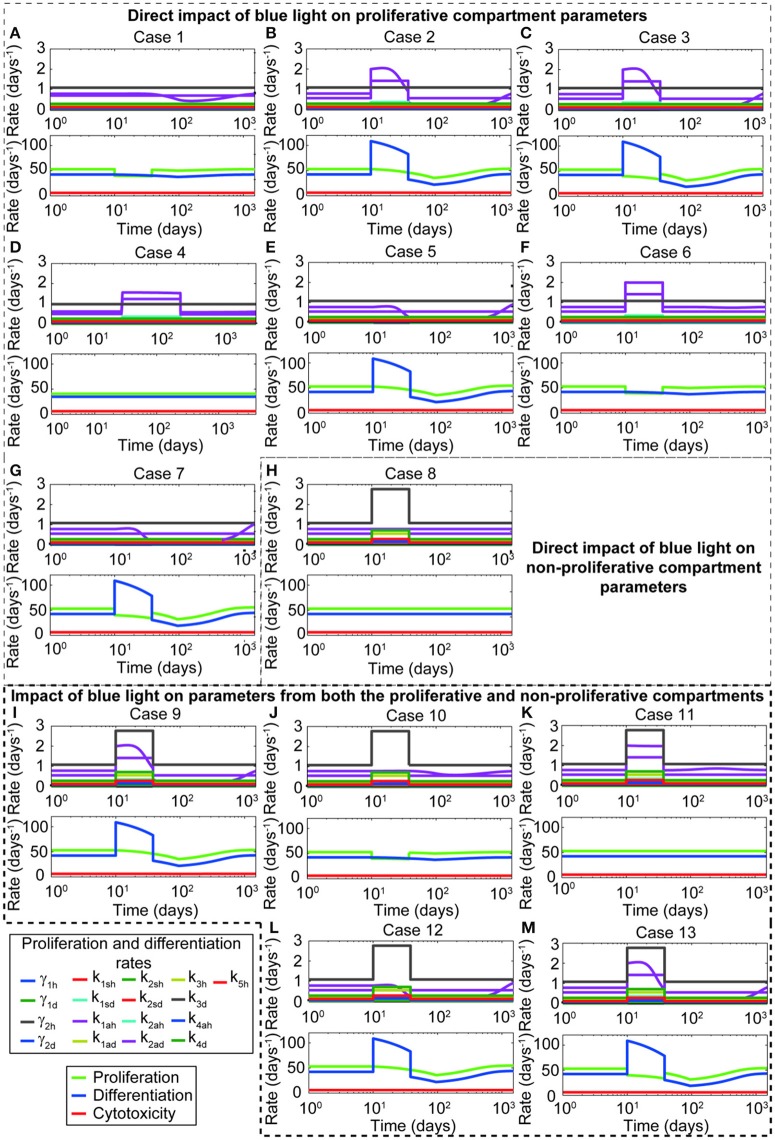
**Representing ***in silico*** the blue light effect by decreasing the proliferation rate of keratinocytes yields the behavior observed experimentally**. The panels show the changes on the proliferation and differentiation of healthy and diseased keratinocytes due to different approaches of blue light *in silico* representation. Thirteen different cases are depicted in this figure. A detailed description of each case can be found in Table 4. These cases are grouped according to the parameters affected by blue light. Panels **(A–G)** show cases where only the proliferation and division parameters from the proliferative compartment are modified during blue light irradiation. Panel **(H)** presents the case where only the differentiation parameters from the non-proliferative compartment are affected. Panels **(I–M)** described the cases where blue light irradiation impacts both compartments. The upper plots of each panel show the impact of the blue light factor on the proliferation and differentiation rates of all keratinocyte populations as a consequence of the parameters affected by blue light. The lower panels display the impact of each potential representation on the proliferative (green), differentiation (blue), and immune system-cytotoxic (red) capacities.

The general set of ODEs used in this analysis is presented by (Equations 1–17). Note that for cases where only θ_*BLγ*_ is considered, θ_*BLk*_ is equal to 1. Similarly when only θ_*BLk*_ is considered, θ_*BLγ*_ equals 1. For cases where only the stem cells and transit amplifying cells are affected, θ_*BLk*_ for the differentiation rates of growth arrested, spinous, and granular cells equals 1. For each case, the time evolution of proliferation and differentiation parameters, the net proliferative and differentiation capacity of the keratinocytes, and the regulatory capacity of the immune system is computed (Figure 2). From this analysis, it is clear that shifting only parameters related to the symmetric and asymmetric division of proliferative keratinocytes does not yield a decrease on the proliferative compartment (Figures 2B,D,E). Further, changing the division parameters causes a marked increase in the differentiation rates (Figures 2B–D,F, H–M), which is reflected in an increased cell density of the transit amplifying cells in the proliferative compartment and all cells in the non-proliferative compartment. Modifying the self-proliferation rate of the proliferative keratinocytes leads to a decrease on the proliferation and increase on the differentiation of the cells in the proliferative compartment, without unrealistic surge of the keratinocytes differentiation capacity (Figure 2A). Thus, we conclude that the effect of blue light is best described *in silico* when θ_*BLγ*_ is lower than 1 and θ_*BLk*_ is equal to 1. The simulations and results presented in the following sections of this work are based on this conclusion.

### A transient decrease in the proliferative capacity of keratinocytes lowers the severity of psoriatic lesions

**Figure 3:**
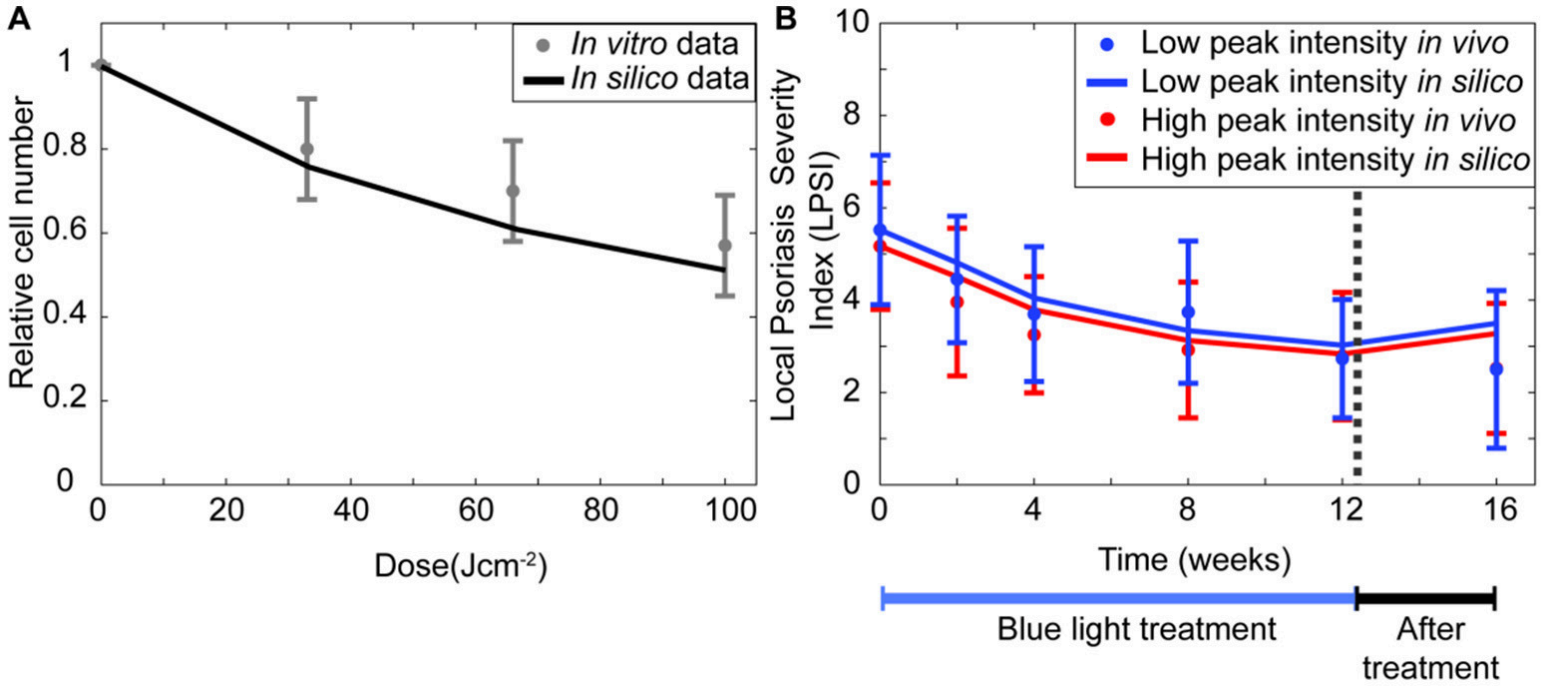
**BLISS accurately describes ***in vitro*** and ***in vivo data***. (A)** The relative keratinocyte density is computed (solid black line) for irradiation schemes with fluences between 0 and 100 Jcm^−2^ and compared to the experimental data (Liebmann et al., 2010) of keratinocytes irradiated with three fluences of continuous wave blue light (gray dot). **(B)** The changes in the severity of a psoriasis lesion during (blue bar) and after treatment (black bar) are predicted (solid lines) for a pulsed wave treatment at high (red) and low (blue) intensities. These changes in the severity of the disease are shown in terms of the local psoriasis severity index (LPSI). The *in silico* results are compared to the clinical data (dots) of Pfaff et al. (2015).

The results from Section The Regulation of the Keratinocytes Proliferation is Key in the Mechanism of Blue Light Irradiation suggested that the experimental observations for keratinocytes (Liebmann et al., 2010) are best described by the model when the self-proliferation rate of all stem cells and transit amplifying cells decreases during blue light irradiation. Simulations performed for the same conditions of that *in vitro* study show a similar reduction in the relative cell number achieved after BL irradiation at fluence of 33, 66, and 100 Jcm−2 (Figure 3A). To verify that the model was also able to describe the reduced severity achieved by blue light treatment of psoriatic skin, simulations (Figure 3B) were performed and compared to data from the clinical investigation of Pfaff et al. (2015). Their clinical study measured the LPSI of psoriatic patients after irradiating the skin with 90 Jcm−2 of pulsed blue light for 12 weeks at either a low (LI), 100 mWcm−2, or high (HI), 200 mWcm−2, peak intensities. During the first 4 weeks the patients were treated every day, the next 8 weeks the therapy occurred three times per week. In the simulations, the initial LPSI values are equal to those of the clinical investigation (Pfaff et al., 2015; i.e., 5.52 for LI and 5.17 for HI). The model achieved an accurate representation of the applied treatments at low and high peak intensities, particularly during the first 4 weeks. In the next 8 weeks, the *in silico* results are less pronounced compared to the clinical study. The final values predicted by the model are within the error margins of the experimental results. Based on the results of the simulations performed for the experimental and clinical studies, it is evident that the transient decrease in proliferation has a pivotal role in the underlying mechanism of blue light treatment.

**Figure 4:**
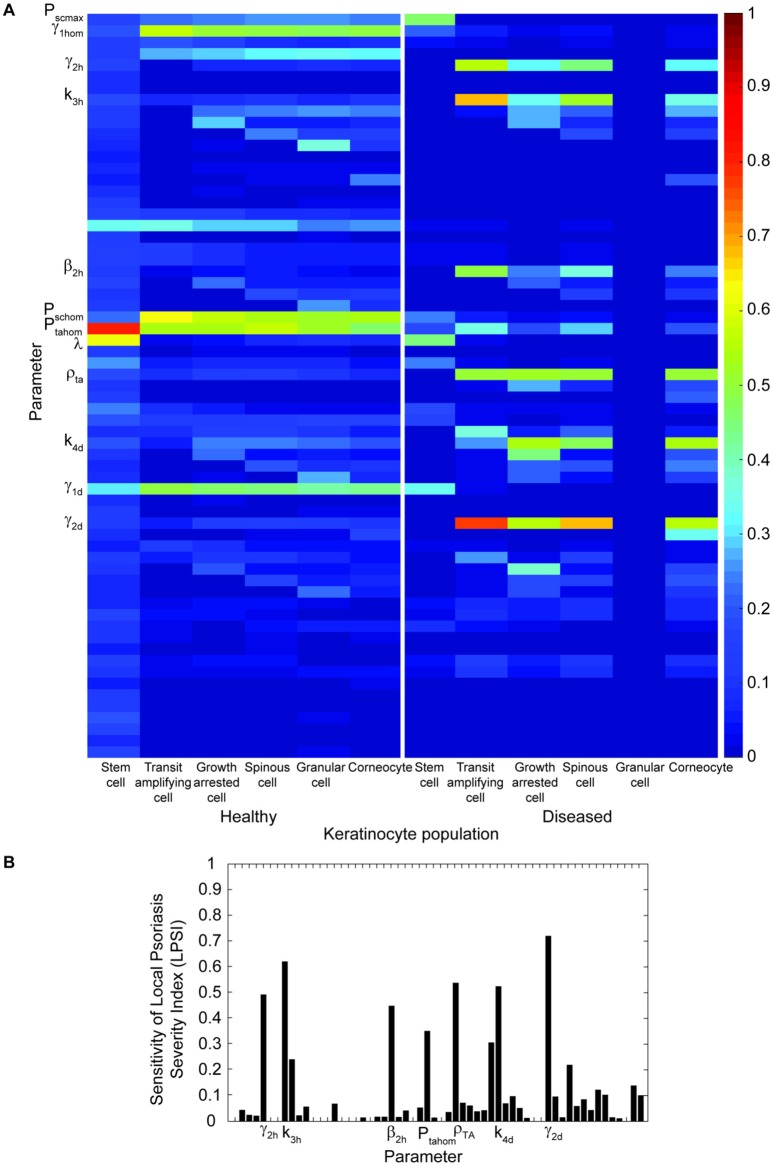
**Multiple parameter sensitivity shows that variations in the proliferation parameters have a high impact on the final keratinocyte cell densities and the local severity of psoriasis at the end of treatment. (A)** The sensitivity of healthy and diseased keratinocytes (x axis) to variations in the 58 model parameters (y axis) is depicted in the heat map. Only those with a Kolmogorov–Smirnov statistic value higher than 0.3 are shown in the y axis. **(B)** The sensitivity of the treatment outcome to changes in the same model parameters of Panel **(A)** are shown in the bar plot in terms of the local psoriasis severity index. Only those parameters with a Kolmogorov-Smirnov statistic value higher than 0.3 are shown in the x axis.

To assess the impact of changes in the proliferation rate and other model parameters on the outcome of the treatment, a multiple parameter sensitivity analysis was implemented. Figure 4 presents the results of this analysis for all model parameters in relation to the keratinocyte cell densities (Figure 4A) and the LPSI (Figure 4B). According to Figure 4A, the model output is mainly affected by 12 parameters related to the proliferation of stem cells and transit amplifying cells. However, not all keratinocyte populations are evenly altered by variations in these parameters, some affect only the healthy populations while others have an impact on the diseased populations. The cell density of psoriatic keratinocytes is mainly affected by the proliferation rate of healthy and diseased transit amplifying cells. Conversely, the proliferation rate of psoriatic stem cells only impacts the healthy populations and the psoriatic stem cells. The LPSA performed for these 12 sensitive parameters indicated that diseased transit amplifying cells are most sensitive to small perturbations on the proliferation rate of healthy transit amplifying cells. This parameter as well as the proliferation rate of diseased stem cells and transit amplifying cells are directly related to the fluence, intensity and irradiation time used by the implemented treatment. From the 12 parameters identified in the MPSA for keratinocytes cell density, only seven have a strong impact on the local severity of psoriasis at the end of treatment (Figure 4B). These seven parameters correspond to those affecting the cell densities of diseased keratinocyte populations. The impact of these parameters on the LPSI is consistent with the effect on the cell density of diseased keratinocytes, but lower in comparison to the latter.

**Figure 5:**
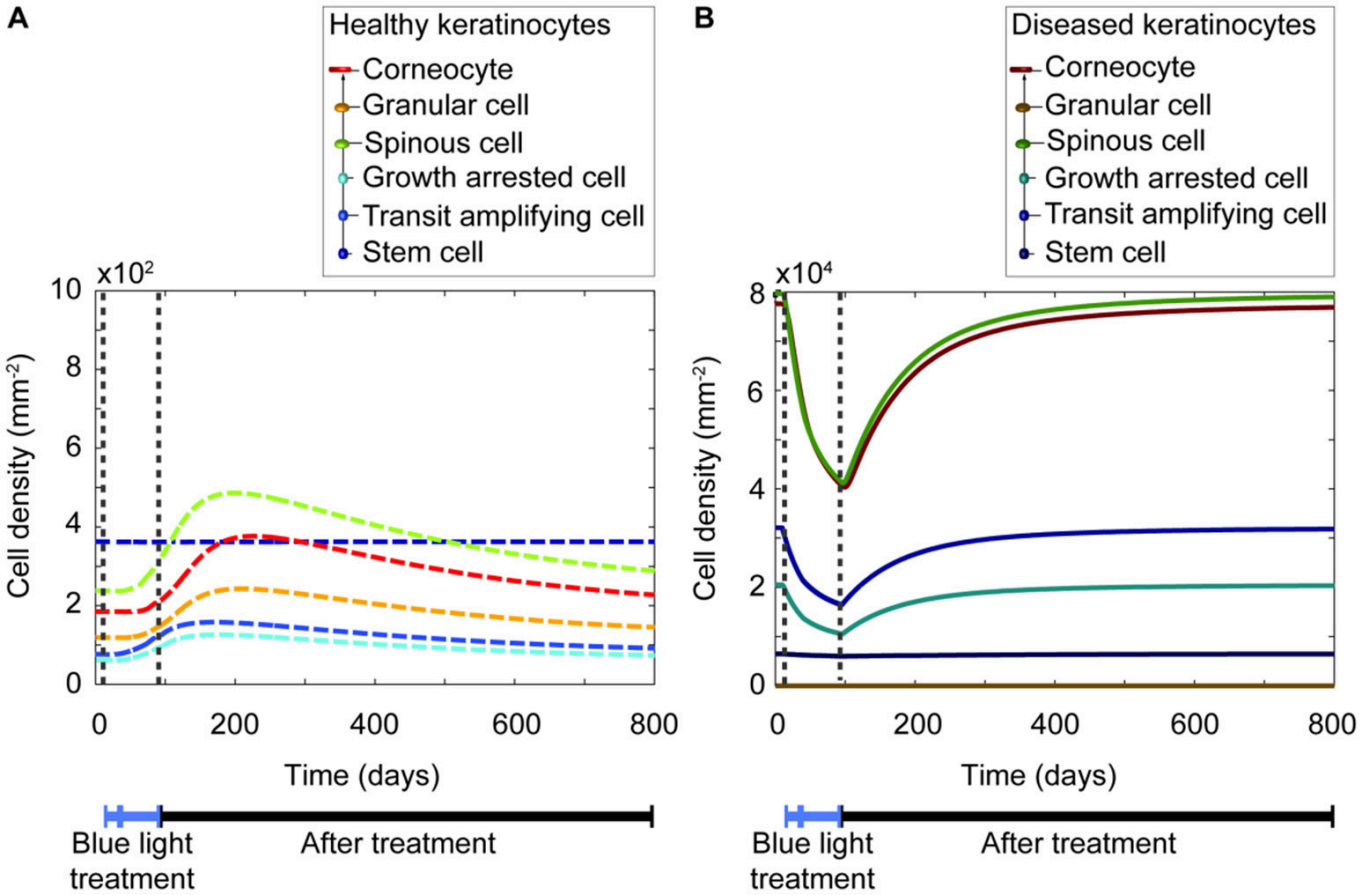
**Blue light induces a temporary reduction of all keratinocyte populations during the length of the treatment. (A)** The time evolution of the healthy keratinocyte cell densities (dashed lines) comprised in a psoriatic epidermis are described during (blue bar) and after (black bar) blue light-based therapy. **(B)** Similarly, the time evolution of the diseased keratinocytes (continuous lines) is shown during and after treatment. Note the difference in scales of the y-axes between panels **(A)** and **(B)**. In this simulation, the treatment periods divided into two sections. The first one consists of 28 days, with irradiation sessions occurring every day. The second section comprises 56 days, with treatment sessions happening three times per week. On each session a fluence of 90 Jcm^−2^ is applied for 30 min.

Based on the sensitivity analysis, it was confirmed that the decreased proliferation capacity induced by blue light irradiation directly impacts all keratinocytes populations. Despite the insightful information this analysis provided, it was yet unclear how each keratinocyte population varied before, during, and after shining blue light on the skin. Thus, a simulation (Figure 5) was executed for the blue light treatment of a lesional skin area of 1 mm^2^ for 84 days, using a fluence of 90 Jcm^−2^. The simulated scheme was divided into two sections, the first one with everyday treatment, and the second one with three times per week irradiations. The time evolution of both healthy and diseased keratinocytes is depicted in Figures 5A,B respectively. The model predicted that the cell density of all keratinocyte populations decreases due to the repetitive irradiation with blue light. However, the cell density of diseased keratinocytes remained considerably high after the treatment compared to the cell density of healthy keratinocytes. Thus, no shift to a healthy phenotype was achieved by this therapeutic approach. The model suggested that a lesional state may prevail after the treatment, but some improvements might be observed within the period of phototherapy. Additionally, it predicted that a less pronounced decrease in the cell density of all keratinocytes is achieved when the treatment occurs every other day compared to daily treatment. This observation suggests that the longer the period of daily treatment the lower the final LPSI value.

### Blue light treatment schemes with long duration and high fluence yield high efficacy in the management of psoriasis

**Figure 6:**
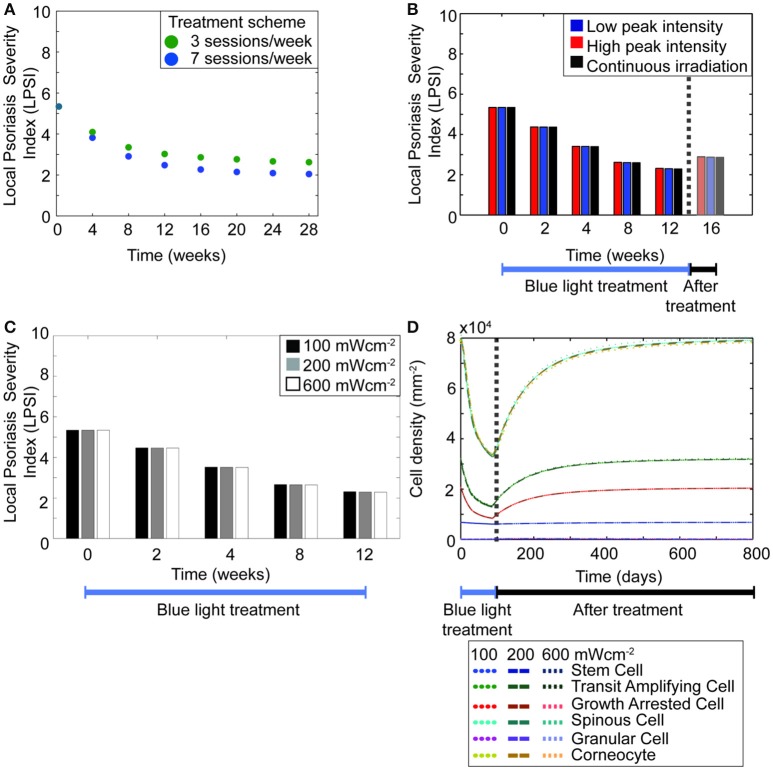
**The length of treatment is a key factor to achieve high treatment efficacy. (A)** The LPSI at the end of treatment is predicted for treatment protocols with a total duration of 4–28 weeks. The treatment sessions in the simulated protocols occur either on a daily (blue dots) or every other day (green dots) basis. **(B)** A 12 weeks treatment with fluence of 90 Jcm^−2^ is simulated for continuous (black) and pulsed irradiation with low (blue) and high (red) peak intensities. **(C)** The variation in the LPSI value is predicted for irradiation schemes with low and high intensities of continuous wave blue light. **(D)** The evolution of the total keratinocyte cell densities during (blue bar) and after (black bar) treatment are derived using the same low and high intensities of continuous wave blue light presented in panel **(C)**.

Hitherto, the model predicted that regulating the hyper-proliferative populations of keratinocytes induces a transient management of psoriasis. However, it also showed that the treatment scheme used in the simulations has a direct impact in the predicted outcome. This observation agrees with the large variations perceived in the results from the available clinical investigations (Maari et al., 2003; Weinstabl et al., 2011; Kleinpenning et al., 2012; Pfaff et al., 2015). Therefore, we studied the impact of each treatment parameter on the efficacy of the therapeutic approach. Figure 5B shows a prominent decrease in the cell densities of diseased keratinocytes when the blue light is constantly shinned upon the skin compared to when the irradiation is less frequent. To further analyze this observation, simulations were executed for therapy schemes with a total duration of 4–28 weeks, using either a daily or every other day treatment (Figure 6A). The results showed that the longer the period of daily treatment the lower the final LPSI value. Nevertheless, the relative change in the LPSI value decreased for treatment periods longer than 20 weeks. Further, BLISS predicted that for a blue light phototherapy scheme where both daily and every other day treatment sessions are used, higher efficacy is achieved with an increasing number of daily treatment sessions.

Given that repeated treatment sessions seemed to yield higher efficacy, we then tested whether the same trend applied to the irradiation mode of treatment scheme. As previously mentioned a given fluence of blue light can be shinned on the skin in either a continuous or pulsed mode. Both cases comprise the same average intensity, however the peak intensity differs. Pfaff et al. studied *in vivo* the impact of low and high peak intensities in the pulsed irradiation of psoriatic skin (Pfaff et al., 2015). They observed minor differences between low and high peak intensity groups; however this trend was not seen *in silico* (Figure 3B solid lines vs. dots). In the model, both low and high intensity pulsed treatment led to the same trend. These results led to questioning whether the model could predict a different outcome for treatment strategies with pulsed and continuous irradiation. Thus, simulations were implemented for low and high power densities in pulsed and continuous modes (Figures 6B–D), considering a 12 weeks treatment and 4 weeks follow-up with an average fluence of 90 Jcm^−2^ and an average intensity of 50 mWcm^−2^ for both irradiation modes. Figure 6B compares the efficacy of pulsed and continuous mode in terms of the local psoriasis severity index. In the simulations for the pulsed mode, low (100 mWcm^−2^) and high (200mWcm^−2^) peak intensities were used. No differences were observed between both modes, or between low and high peak power densities. Hence, the model suggested that duration rather than intensity determines the efficacy of the treatment. This prediction was further tested by simulating the treatment of a psoriatic epidermis using power densities of 100, 200, and 600 mWcm^−2^ in the continuous mode (Figures 6C,D).

**Figure 7:**
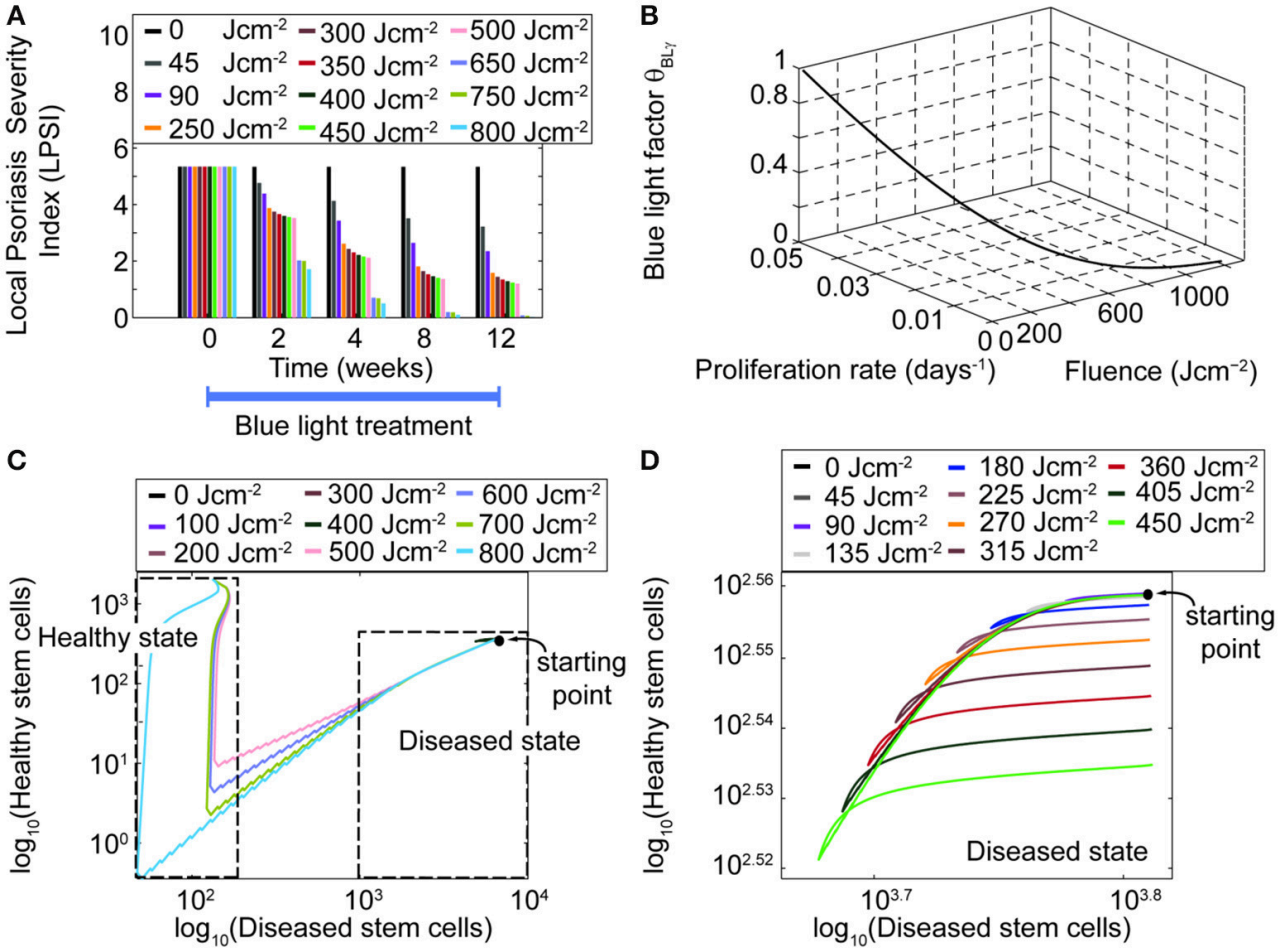
**High fluences of blue light yield higher management of psoriasis than low fluences. (A)** The LPSI over time is computed for a 12 weeks continuous wave treatment with an initial LPSI of 5.17 (Pfaff et al., 2015) using 11 fluences. **(B)** The behavior of the blue light factor θ_*BLγ*_ and the proliferation rate are visualized as functions of the fluence. **(C)** The bi-stability of the model is evaluated in the logarithmic scale for a wide range of fluences in a phase diagram of healthy and diseased keratinocytes. Only fluences above 500 Jcm^−2^ yield a shift the dominant keratinocyte population and the phenotype of the skin. **(D)** Fluences below this level remain in the disease state.

In addition to time and intensity, fluence is another important parameter of the treatment scheme. Simulations performed for fluences between 0 and 750 Jcm^−2^, considering a treatment of 12 weeks (Figure 7A) predicted that fluence levels as low as 45 Jcm^−2^ lead to a decrease in the LPSI value. Moreover, at fluence levels between 200 and 500 Jcm^−2^ a saturation point was observed. At fluences equal or higher than 500 Jcm^−2^ the decrease in the LPSI value was considerably larger due to the cytotoxicity induced by BL at these levels. According to these results, low fluences would lead to a minor decrease in the LPSI compared to higher fluences. This observation is better understood when analyzing the relation between the fluence, the blue light factor θ_*BLγ*_, and the consequent proliferation rate derived by the model (Figure 7B). Here, we show only the analysis for the proliferation rate of diseased transit amplifying cells, but the trends are consistent for the other proliferative populations. A clear exponential decrease is seen on both the blue light factor and the proliferation rate with increasing fluences. However, the impact of fluence on the proliferation rate is steeper and faster compared to the effect on the blue light factor. Further, for fluences below 500 Jcm^−2^ both the blue light factor and the proliferation rate are reduce in an almost linear manner. But it is less abrupt at fluences above 200 Jcm^−2^. The fluence effect translates into a linear reduction of the proliferation in relation to the blue light factor, which yields a lower cell density and the consequent improvement on the psoriatic skin. The model was designed to allow bi-stability at either a diseased or healthy phenotype. The healthy state can only be reached when the total cell density at the end of treatment is dominated by healthy keratinocyte populations. To achieve the switch in the ruling type of population, the number of diseased cells must be considerably lowered during the treatment. Our results showed that this shift in phenotypes could not be acquired with blue light fluences where no cytotoxicity was induced (Figures 7C,D). Figure 7C presents the relation between healthy and diseased stem cells with increasing fluence levels. From this figure is clear that only fluences above 500 Jcm-2 achieve a majority of healthy stem cells. Fluences below that level reduce the cell densities but remain within the diseased state due to a high predominance of diseased keratinocytes (Figure 7D).

## Discussion

Blue light at 420 and 453 nm is highly effective in the management of psoriasis (Weinstabl et al., 2011; Pfaff et al., 2015) without any adverse effects (Awakowicz et al., 2009; Kleinpenning et al., 2010). We have explored the underlying mechanism of this therapeutic method and the impact of the treatment scheme on the efficacy of the treatment using an *in silico* approach. BLISS suggests that the decrease in the keratinocytes proliferative capacity is sufficient to induce a transient decrease in the severity of psoriasis. Further, it predicts that treatment schemes with long duration and high fluence yield the most optimal outcome. The model constitutes a fast and flexible tool that considers not only the properties of the irradiated epidermis but also the interactions between the structural cells within it. Our *in silico* method provides a framework for further insight on the underlying mechanism of this blue light-based phototherapy and the optimal treatment of psoriasis. To the extent of our knowledge, this is the first model that studies the changes induced by blue light irradiation on the cell dynamics of keratinocytes. The general concepts of epidermal kinetics used here have been applied to UV phototherapy (Grabe and Neuber, 2007; Weatherhead et al., 2011; Zhang et al., 2015) before. Nevertheless, the differences on the underlying mechanism make the previous models and ours two utterly different applications.

On this work, we first addressed how to best represent these phenomenological observations *in silico* while accounting for the characteristic phenotype of psoriasis (Perera et al., 2012). From experimental studies, it is known that blue light decreases the proliferative capacity of keratinocytes and other cell types (Taoufik et al., 2008; Wataha et al., 2008), and that it reduces their proliferation by inducing their differentiation (Liebmann et al., 2010). However, it is not clear how exactly these changes are exerted on the cellular processes of keratinocytes. BLISS accounts for the three main theories of cell division (Clayton et al., 2007; Klein et al., 2011; Watt, 2014), i.e., self-proliferation, asymmetric, and symmetric division. Self-proliferation refers to the ability of a cell dividing into two daughter cells of the same kind. Asymmetric division considers that one proliferative cell divides intro one daughter cell of the same kind and one of the next differentiation stage. Finally, symmetric division comprises the division of a proliferative cell into two cells of the next differentiation stage. In the model the proliferation and differentiation of keratinocytes in the proliferative compartment is represented by their self-proliferation, symmetric, and asymmetric division parameters. The keen reader could think that in order to best describe the effect of blue light on proliferation and differentiation, increasing the rates of asymmetric and symmetric division should be enough to achieve similar observations as those reported *in vitro*. Alternatively, the direct increase on the division rates of proliferative cells should be coupled to the decrease of the self-proliferation rate of all keratinocytes in the proliferative compartment. Thus, capturing the increase in differentiation and the decrease in proliferation of the irradiated keratinocytes. Our analysis (Figure 2) showed that modifying the division rates alone does not appropriately reflect on the proliferative capacity of the cells. Additionally, it translates into high peaks in the cell density of transit amplifying and non-proliferative cells, which are biologically unlikely. However, decreasing only the proliferation rate does yield the effect on the differentiation and proliferative capacity of keratinocytes observed experimentally. The outcome of this approach is a transient decline in the proliferative rates of the cells and consequent higher differentiation, translated into a temporary drop local severity of the disease. BLISS comprises a fine tuning between the proliferation and division rates of all keratinocytes, particularly of healthy stem cells. It considers a strong connection between the proliferation and division rates of healthy stem cells and their regulation by the total population of healthy and diseased transit amplifying cells. This fact might explain the outcome of our analysis. From the sensitivity analysis (Figure 4), it was clear that proliferation-related parameters had the highest influence on the final cell densities and the improvement of psoriasis due to blue light treatment. The most sensitive parameters were directly related to the treatment scheme. Their impact was reflected on the cell density of diseased keratinocyte populations and local psoriasis severity index. These observations may explain the large variations observed among clinical investigations of BL treatment (Maari et al., 2003; Weinstabl et al., 2011; Pfaff et al., 2015).

Previous models of phototherapy, particularly UV-based approaches represented the therapeutic effect by inducing strong apoptosis on the keratinocytes (Weatherhead et al., 2013; Zhang et al., 2015). This temporal increase in the apoptotic rate leads to a remission phase, characterized by a lower total cell density after the treatment compared to the initial one. In our model, apoptosis is only affected at fluences above 500 Jcm^−2^, instead the proliferation and differentiation capacity of keratinocytes are altered during the treatment sessions. The key difference in the approaches of representing UV and BL phototherapy *in silico* is defined by the difference in the underlying mechanisms of both therapeutic approaches. Despite both methods affecting the cells behavior by absorption of light at a certain wavelength, the photo-acceptors and consequent events differ. UV is mainly absorbed by the cell's DNA (Benedix et al., 2005), consequently inducing direct DNA damage, apoptosis (Weatherhead et al., 2011), and a decrease in the total cell density of the lesional skin. Blue light irradiation does not induce DNA damage (Awakowicz et al., 2009), it is absorbed by other photo-acceptors in the cell, e.g., flavins (Sadeghian et al., 2008) and cryptochromes (Bouly et al., 2007). The consequent effect is the release of nitric oxide (Opländer et al., 2013) and reactive oxygen species (Yoshida et al., 2013), which alter the cell's proliferation and differentiation rates.

Clinically, this transient effect on the keratinocytes proliferation and differentiation is observed as the improvement of a psoriatic lesion (Weinstabl et al., 2011; Kleinpenning et al., 2012). The model is bi-stable, thus it can reproduce steady states for both healthy and diseased skin phenotypes. In our simulations, we account for mild psoriasis through a specific set of initial cell densities and show that although the plaque is controlled during the treatment the underlying state is still diseased. After the treatment, the model eventually goes back to the initial diseased state. In the UV phototherapy model of Zhang et al. (2015), they demonstrate that UV treatment with consecutive episodes can theoretically control the disease and achieve a remission of the psoriatic phenotype. In our model, we do not observed this remission phase for mild psoriasis considered with fluences below 500 Jcm^−2^.The lack of remission phase in our simulations for blue light treatment in contrast with UV phototherapy may be due to the underlying mechanism. Increasing the apoptosis of keratinocytes yields a lower cell density regardless of their proliferation and differentiation rate. This effect results in a shift of the stem cell density from the disease state to the healthy state (Figure 7C). Altering the proliferation and differentiation rates of the proliferative keratinocytes due to blue light irradiation only impacts their apoptosis rates at high fluence levels. Thus, the effect of blue light is mild compared to that of UV (Weatherhead et al., 2013). Nevertheless, it is possible that other factors are involved in the underlying mechanism of blue light management of psoriasis. The inclusion of these factors in the model could potentially yield to the shift in phenotypes and full remission of psoriatic skin. In our method, the immune system regulates the dynamics of the keratinocytes through the removal of disease stem cells at a rate defined by the maximum immune killing rate and the half activation of the immune system (Equation 7). Blue light also induces apoptosis of T-cells (Liebmann et al., 2010; Oh et al., 2016), alter the immune response (Fischer et al., 2013) and decrease inflammation (Shnitkind et al., 2006). However, the impact of BL on the immune system is not considered in the current model. The inclusion of blue light related effects on the immune system might result in lower keratinocyte cell densities and the consequent shift in the phenotype. The available clinical investigations have not assessed yet the impact of blue light on the immune system in the context of psoriasis. Data from skin biopsies, experimental, and clinical studies on blue light irradiation is needed to determine which aspects could be involved and how they should be included in the model.

BLISS explicitly includes relevant treatment parameters, i.e., fluence, time, intensity, and mode of irradiation. This feature of the model allows us to investigate the influence of these parameters on the management of psoriasis. Recent experimental studies have explored the effect of various wavelengths on the blue range of the electromagnetic spectrum (Liebmann et al., 2010; Opländer et al., 2013), and fluence on various cell types (Awakowicz et al., 2009; Liebmann et al., 2010; Sparsa et al., 2010; Monfrecola et al., 2014). Clinically, the limited number of available studies focuses on the assessment of treatment efficacy with a specific combination of parameters. Only two studies implements a wavelength comparison (Weinstabl et al., 2011; Kleinpenning et al., 2012), and another evaluates high and low pulsed intensities (Pfaff et al., 2015). Nevertheless, no global guidelines for treatment have been defined. Furthermore, the impact on the kinetics of the main cells involved in psoriasis and their role on the management of the disease are yet to be resolved. One of the objectives of this study was to study the impact of treatment parameters on the blue light mediated regulation of keratinocytes in psoriasis and the consequent control of the lesion. Our results suggested that therapy length and the applied fluence rather than intensity of irradiation may potentially determine the efficacy of the treatment. Simulations for various treatment lengths (Figure 6A) showed that algorithms with a long treatment period and consecutive episodes may be the most effective for psoriasis. This observation could partially explain the higher improvement of Pv symptoms achieved by Pfaff et al. (2015) (12 weeks) in contrast with prior investigations (4 weeks; Maari et al., 2003; Weinstabl et al., 2011). Another reason could be the different fluences used in these studies [10 Jcm^−2^ (Maari et al., 2003) and 90 Jcm^−2^ (Weinstabl et al., 2011; Pfaff et al., 2015)], given the fluence dependency associated with blue light irradiation (Liebmann et al., 2010; Dai et al., 2012). Simulations implemented for a wide range of fluences (Figure 7) show a negative correlation between fluence and final LPSI value. However, no threshold value has yet been defined. To define this value additional *in vitro* and *in vivo* data are needed. Research on light therapy for different applications has indicated that pulsed light is more effective than continuous light, while others report no effect, or a worsening effect of pulsed irradiation compared to no treatment (Lapchak et al., 2007; Hashmi et al., 2010). In Psoriasis, only one clinical investigation has used a pulsed irradiation mode (Pfaff et al., 2015), while the others employed the continuous mode (Maari et al., 2003; Weinstabl et al., 2011; Kleinpenning et al., 2012). Simulations performed with the model (Figure 6B) indicated that pulsed light is as effective as continuous light in the treatment of psoriasis.

The current model has some addressable limitations: (i) it does not explicitly comprise the photo-activated processes leading to the cellular regulation and management of psoriasis. (ii) We neglected the additional cytotoxicity induced on the keratinocytes at wavelengths shorter than 453 nm (Liebmann et al., 2010). The model was built assuming irradiation at 453 nm, which is the most common wavelength used for psoriasis (Weinstabl et al., 2011; Pfaff et al., 2015). Despite the minimum differences in treatment efficacy reported in the literature (Kleinpenning et al., 2010; Weinstabl et al., 2011), inclusion of this effect could be worth a thorough analysis. However, additional data is needed on the optical parameters and the skin before this feature can be included in the model.

One definite experimental test of our model should aim at simultaneously tracing psoriasis and healthy proliferative and non-proliferative keratinocytes to verify whether blue light equally affects healthy and diseased proliferative cells. One potential approach to test this hypothesis could be isolating keratinocytes from psoriatic skin (Aasen and Belmonte, 2010) after shinning blue light on it. In this work, we provide general insights and recommendations based on the model results. In the future, more precise recommendations could be derived from the model by implementing an optimization analysis for the treatment (Kessel et al., 2013). Our results showed that length of treatment is vital in decreasing the severity of a psoriasis lesion, however there is little information reported in the literature on the direct effect of irradiation length and cumulative exposure of cells to blue light. Future studies should explore this theory experimentally to define the boundaries of the cellular response and the most optimal outcome. Also, they should assess the long term management of psoriasis and the recurrence of the lesions.

## Conclusions

Overall, this study demonstrates that regulation of the proliferative capacity of keratinocytes is a crucial process in the blue light induced management of psoriasis. Considering the uprising interest in blue light as treatment for inflammatory skin conditions, the availability of irradiation guidelines becomes of great relevance. Our model constitutes a flexible tool for studying the underlying mechanism, formulating valuable recommendations, and designing effective therapeutic regimes with BL in a constraint-free environment.

## Author contributions

Wrote the paper: ZCFG. Conceived and designed the computational framework: ZCFG, JL, MB, PAJH, NAWvR. Developed the model: ZCFG. Performed the computational analysis: ZCFG. Revised the paper: JL, MB, PAJH, NAWvR. Supervised the study: JL, MB, PAJH, NAWvR.

## Funding

This project was funded under the public-private partnership between Philips and the Eindhoven University of Technology.

### Conflict of interest statement

The authors declare that the research was conducted in the absence of any commercial or financial relationships that could be construed as a potential conflict of interest.

## Footnotes

^1^https://github.com/ZFelixGarza/BLISS

^2^http://www.ebi.ac.uk/biomodels-main/

## Abbreviations

BL: Blue Light
Pv: Psoriasis vulgaris
F: Fluence
UV: Ultraviolet light
BLISS: Computational model for Blue Light Irradiation of Psoriatic Skin
P_sc_: Stem Cells
P_ta_: Transit Amplifying cells
P_ga_: Growth Arrested cells
P_sp_: Spinous cells
P_gc_: Granular Cells
P_cc_: Corneocytes
I_av_: Average power density
CW: Continuous Wave mode
PW: Pulsed Wave mode
I_p_: Peak power density
PASI: Psoriasis Area and Severity Index
LPSI: Local Psoriasis Severity Index
MPSA: Multiple parameter sensitivity analysis
LPSA: Local sensitivity analysis
LI: Low Intensity
HI: High Intensity.
